# Synergistic augmentation and fundamental mechanistic exploration of β-Ga_2_O_3_-rGO photocatalyst for efficient CO_2_ reduction†

**DOI:** 10.1039/d4na00408f

**Published:** 2024-07-15

**Authors:** Hye-In Jung, Hangyeol Choi, Yu-Jin Song, Jung Han Kim, Yohan Yoon

**Affiliations:** a Korea Aerospace University, Department of Materials Engineering Goyang Republic of Korea yyoon@kau.ac.kr; b Dong-A University, Department of Materials Science and Engineering Busan Republic of Korea

## Abstract

We explore the novel photodecomposition capabilities of β-Ga_2_O_3_ when augmented with reduced graphene oxide (rGO). Employing real-time spectroscopy, this study unveils the sophisticated mechanisms of photodecomposition, identifying an optimal 1 wt% β-Ga_2_O_3_-rGO ratio that substantially elevates the degradation efficiency of Methylene Blue (MB). Our findings illuminate a direct relationship between the photocatalyst's composition and its performance, with the quantity of rGO synthesis notably influencing the catalyst's morphology and consequently, its photodegradation potency. The 1 wt% β-Ga_2_O_3_-rGO composition stands out in its class, showing a notable 4.7-fold increase in CO production over pristine β-Ga_2_O_3_ and achieving CO selectivity above 98%. This remarkable performance is a testament to the significant improvements rendered by our novel rGO integration technique. Such promising results highlight the potential of our custom-designed β-Ga_2_O_3_-rGO photocatalyst for critical environmental applications, representing a substantial leap forward in photocatalytic technology.

## Abstract 2

We explore the novel photodecomposition capabilities of β-Ga_2_O_3_ when augmented with reduced graphene oxide (rGO).

## Graphical abstract

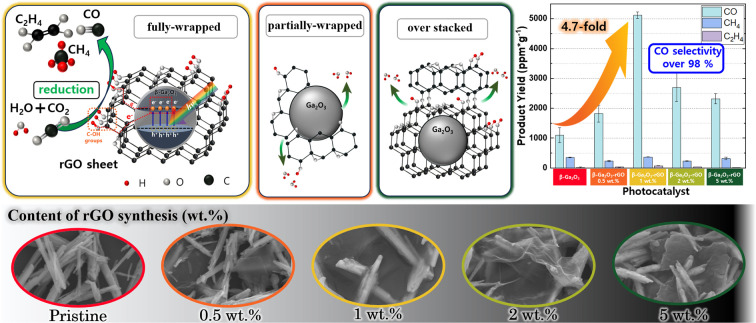

## 1. Introduction

As technology continuously advances, there is increasing focus on research and development aimed at purifying and decomposing pollutants. The aim of these initiatives is to curb the environmental degradation resulting from these pollutants,^1–4^ an issue that is becoming increasingly critical.^5–7^ Of the numerous strategies currently under exploration,^8–11^ photocatalysis has surfaced as a leading contender in the fight against environmental pollution.^12–18^ This technology, both environmentally friendly and sustainable, taps into the abundant and readily available resource of solar light for pollutant decomposition.^19,20^

Photocatalysts, such as TiO_2_,^21–26^ ZnS,^27–30^ and Ga_2_O_3_,^31–33^ which have been subjects of extensive study, offer the distinct advantages of low energy consumption and the ability to leverage natural sunlight. Among these, Ga_2_O_3_ mirrors TiO_2_ in its oxygen vacancies and has exhibited superior photocatalytic capabilities across a wide spectrum of wavelengths relative to other photocatalysts.^34,35^ Further, Ga_2_O_3_'s high chemical stability enables prolonged reusability,^36–39^ setting it apart from other photocatalysts like TiO_2_ or ZnS. Importantly, its high energy bandgap is particularly effective for CO_2_ decomposition, marking a significant stride in CO_2_ reduction efforts.^8,40–48^

However, despite their promising characteristics, the practical applications of these photocatalysts have been hampered by their suboptimal efficiency in the ultraviolet region.^49^ In response, researchers have begun investigating the use of doping with other elements to enhance their energy absorption capabilities. For example, doping with substances like fluoride,^50^ carbon,^51–53^ and other materials,^54–56^ creating Ga^+3^ and O vacancies (VO) in the doped region, has resulted in the production of photocatalysts that can absorb visible light.^57–59^ In addition, employing hydrothermal synthesis to create a carbon-enabled Ga_2_O_3_-rGO composite not only preserves electron–hole pairs (EHPs) but also boosts their transport capacity.^60–62^ Thus, in this study, our objective is to assess the potential of improved Ga_2_O_3_-rGO photocatalysts in addressing the pressing issue of CO_2_ reduction, a challenge that has been drawing increasing attention.^9,31,62–69^ The photocatalyst employed in our study was synthesized using a hydrothermal method, yielding a nanorod morphology that augments its surface area. By fine-tuning the sintering temperature, we acquired β-Ga_2_O_3_, which demonstrated enhanced photocatalytic performance. We then undertook experiments to establish the optimal concentration of graphene oxide (GO) and the most effective amount of photocatalyst through hydrothermal synthesis to achieve peak photolysis performance.

Nonetheless, given the real-time nature of the photodegradation process, it becomes crucial to explore the degradation mechanism over time. While ultraviolet-visible (UV-vis) spectroscopy provides quantitative insights into photodegradation, their application for *in situ* measurements is challenging, leaving the real-time degradation mechanism largely unexplored. In light of this, we employed a previously developed real-time spectroscopic instrument^70,71^ to rapidly and accurately analyze the Ga_2_O_3_-rGO photocatalyst *in situ* and present the optimized conditions.

## 2. Results and discussion

### 2.1 Transformation and optimization of β-Ga_2_O_3_ nanorods with rGO for enhanced photocatalytic efficiency

**Fig. 1:**
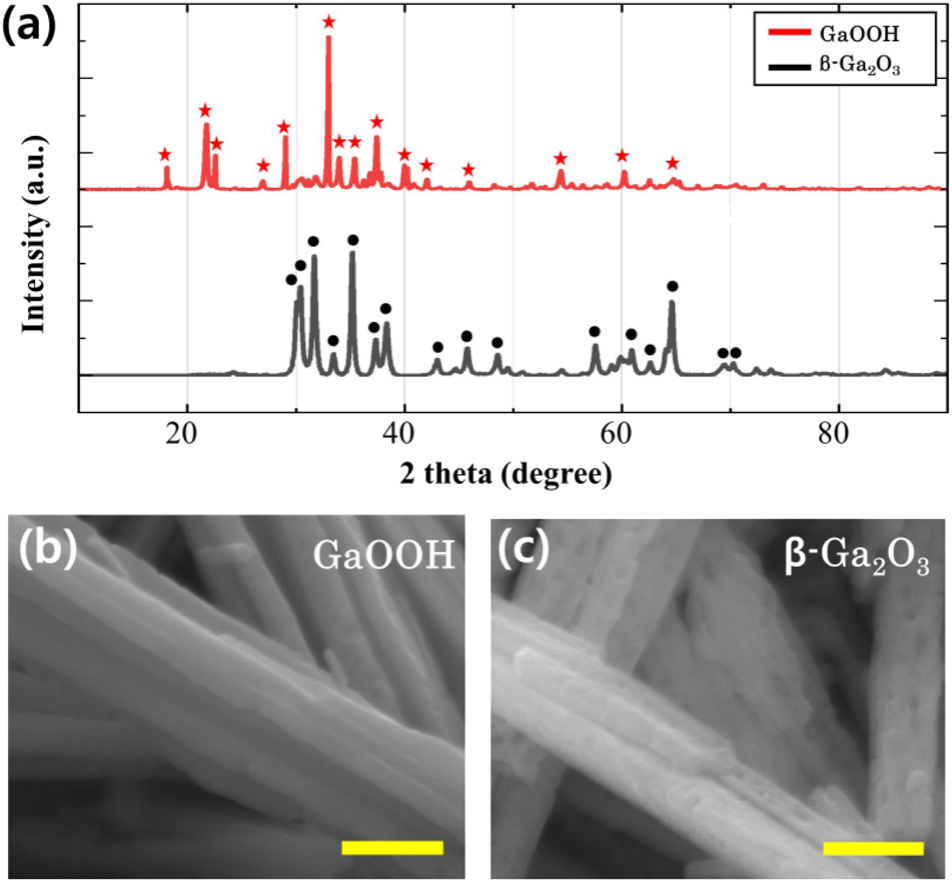
(a) XRD patterns of GaOOH (red stars) and β-Ga_2_O_3_ (black circles). FE-SEM images of (b) GaOOH and (c) β-Ga_2_O_3_ samples obtained during the sintering process. All scale bars are 500 nm.

In this study, phase identification of samples during the sintering process was confirmed using an X-ray diffraction analyzer (XRD). Red stars and black circles in Fig. 1a represent diffraction peaks before and after the sintering process, respectively. Among the red star peaks, the main GaOOH peak with the highest intensity is located at 9.1°, and the other peaks correspond to the GaOOH diffraction peak (JCPDS-0180). After sintering, the 9.1° peak initially observed in the red star peaks disappears, while the most intense peaks are observed at 31.7° and 35.2°, corresponding to the main peaks of β-Ga_2_O_3_. The remaining peaks also align with the diffraction peaks of β-Ga_2_O_3_. These findings indicate that GaOOH was fully converted to β-Ga_2_O_3_ without forming any other substances, suggesting a successful transformation during the sintering process.

Fig. 1b and c present FE-SEM images of GaOOH and β-Ga_2_O_3_, respectively. Both samples exhibit a nanorod morphology, with β-Ga_2_O_3_ displaying a porous structure. This can be attributed to the pH adjustment during nanorod formation and the subsequent removal of portions containing OH groups during the sintering process. Consequently, we employ β-Ga_2_O_3_, a porous nanorod structure with enhanced activity, to develop the β-Ga_2_O_3_-rGO photocatalyst discussed in the following section.

The integration of reduced graphene oxide (rGO) with photocatalysts can significantly enhance photodegradation efficiency.^72–76^ This improved photocatalytic performance can be attributed to the formation of an rGO layer. During the hydrothermal synthesis process, the graphene oxide (GO) layer, created by combining graphene and oxygen, is subjected to heat and pressure, which leads to the loss of oxygen groups and conversion to an rGO layer. The removal of oxygen groups from the GO layer results in an rGO layer where unpaired π electrons are not stabilized. This allows for the formation of π–π bonds as external electrons are absorbed. Consequently, excited electrons from the photocatalyst migrate to the rGO layer and subsequently reduce the bound photolytic material (in this case, MB).^77^ This process enhances efficiency by reducing the bandgap energy of β-Ga_2_O_3_, which has a high bandgap, thereby increasing the energy region absorbed by the photocatalyst to the visible light region and maintaining the EHPs for photodecomposition.^60,61,78,79^

However, the mechanism by which β-Ga_2_O_3_ combines with rGO at different amounts and how this affects photocatalytic efficiency has not been thoroughly investigated. To understand this mechanism, the microstructure was analyzed for a sample set with four different rGO contents and β-Ga_2_O_3_, as mentioned in the experimental section. The objective of this analysis was to determine the optimal ratio between β-Ga_2_O_3_ and rGO. By examining the microstructure using FE-SEM and photocatalytic performance at various β-Ga_2_O_3_ to rGO ratios using real-time spectroscopy in the following section, we can gain insights into the role of rGO in the photocatalytic system. This analysis will help develop a deeper understanding of the underlying mechanisms that contribute to enhanced photocatalytic efficiency.

**Fig. 2:**
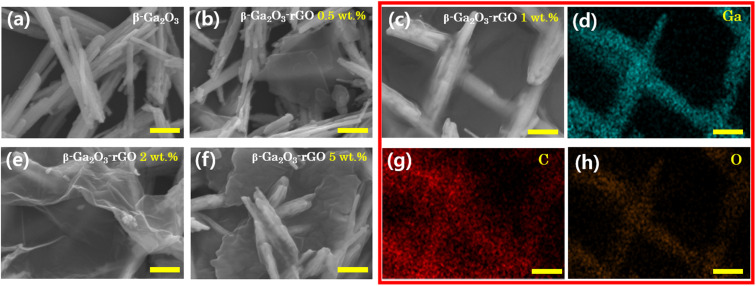
FE-SEM images of (a) β-Ga_2_O_3_ and β-Ga_2_O_3_-rGO composite with various rGO concentrations; (b) 0.5 wt%, (c) 1 wt%, (e) 2 wt% and (f) 5 wt%. EDS mapping of β-Ga_2_O_3_-rGO 1 wt% (c); Ga (d), C (g), O (h). All scale bars are 1 μm.

The morphologies of various samples, including β-Ga_2_O_3_ and β-Ga_2_O_3_-rGO with four distinct rGO contents (0.5 wt%, 1 wt%, 2 wt%, and 5 wt%), synthesized through hydrothermal methods, were investigated using FE-SEM, as depicted in Fig. 2a–c, e, and f. The microstructure of β-Ga_2_O_3_ without rGO (Fig. 2a) displays the rod-like morphology of β-Ga_2_O_3_, as previously observed in Fig. 1c. With the addition of 0.5 wt% rGO, the rGO does not entirely cover the β-Ga_2_O_3_ nanorods' surface, and the majority of rGO appears as separate plates (Fig. 2b). As the rGO content increases to 1 wt%, most of the β-Ga_2_O_3_ nanorods are observed to be wrapped by rGO, as illustrated in Fig. 2c. This observation is further supported by EDS analysis, presented in Fig. 2d, g, and h. The Ga (Gallium) and O (Oxygen) mapping images in Fig. 2d and h clearly depict the nanorod morphology. Additionally, the C (Carbon) mapping image in Fig. 2g confirms that most of the carbon is detected on the nanorods, indicating that the β-Ga_2_O_3_ nanorods are well-wrapped by rGO. As the amount of content increases, rGO tends to aggregate instead of encapsulating. Initially, this results in the formation of folded rGO sheets (as shown in Fig. 2e), which eventually congregate into larger rGO clusters (refer to Fig. 2f). For additional insights into the structural development of the β-Ga_2_O_3_-rGO composite with varying concentrations of rGO, see Fig. S1.† This illustration clarifies the progression from the initial attachment of rGO sheets to their eventual clustering on the β-Ga_2_O_3_ nanorods, demonstrating the influence of rGO quantity on the composite's morphology and its photocatalytic activity.

**Fig. 3:**
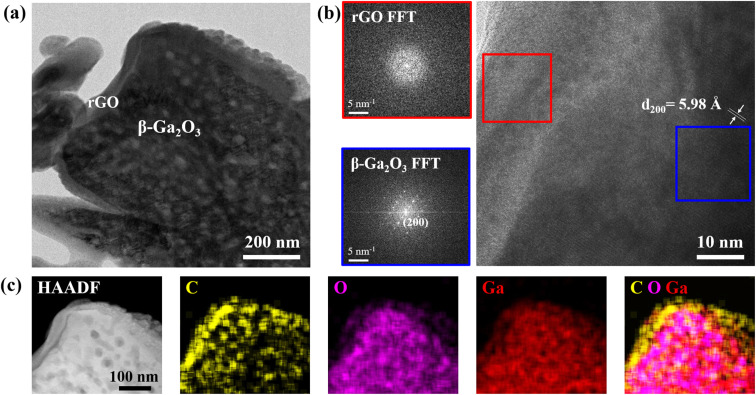
(a) TEM image, (b) HR-TEM image and (c) STEM and elemental mapping images of the β-Ga_2_O_3_-rGO 1 wt% photocatalyst.

TEM analysis, as presented in Fig. 3, offered a closer look into the nano-scale architecture of the β-Ga_2_O_3_-rGO 1 wt% sample. The TEM images at different magnifications disclosed the nanorods being uniformly enshrouded by nanosheets (Fig. 3a). The HR-TEM images, specifically, provided clarity on the crystalline integrity of the nanorods, displaying well-defined lattice fringes with a spacing of 5.98 Å that match the (200) plane of β-Ga_2_O_3_ (Fig. 3b). This precise lattice spacing is pivotal because it confirms the retention of the crystalline structure post synthesis with rGO, a structure that is essential for the photocatalytic activity due to its influence on the electronic band structure.

At the interface of the β-Ga_2_O_3_-rGO 1 wt% sample seen in Fig. 3a, the rGO layer's efficient encapsulation is evident, contrasting with the thicker coatings observed in samples with higher rGO content (2 wt% and 5 wt%) (Fig. S2†). While the conductive rGO is known to facilitate electron–hole pair (EHP) separation due to its exceptional electrical conductivity, the TEM findings suggest that there is an optimal thickness for this rGO layer. Beyond this optimal point, indicated by the increased thickness in higher rGO content samples, there might be a counterproductive effect where excessive rGO could act as an electron–hole recombination center instead of serving as a conduit for charge carrier mobility.^80,81^ This is significant because it suggests a trade-off between the desired increased conductivity and the unintended shielding of reactive sites on the nanorods.

Furthermore, elemental mapping (Fig. 3c) confirmed the composite nature of the sample by showing a distribution of C, O, and Ga across the nanorod surface. This mapping supports the porosity of the β-Ga_2_O_3_, which is crucial for providing a high surface area conducive to photocatalytic reactions, and simultaneously substantiates the successful integration of rGO. The homogeneous presence of carbon in particular points to a uniform rGO coating, which is likely to result in consistent photocatalytic activity across the sample.

**Fig. 4:**
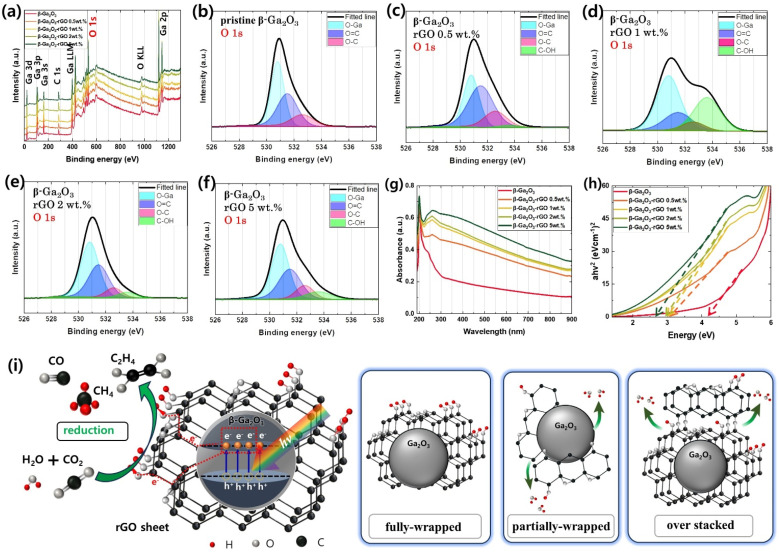
(a) XPS survey spectra of photocatalysts; β-Ga_2_O_3_ (red line), β-Ga_2_O_3_-rGO 0.5 wt% (orange line), β-Ga_2_O_3_-rGO 1 wt% (yellow line), β-Ga_2_O_3_-rGO 2 wt% (light green line), and β-Ga_2_O_3_-rGO 5 wt% (green line). High-resolution XPS O 1s core-level spectra for β- Ga_2_O_3_-based photocatalysts: (b) β-Ga_2_O_3_, (c) β-Ga_2_O_3_-rGO 0.5 wt%, (d) β-Ga_2_O_3_-rGO 1 wt%, (e) β-Ga_2_O_3_-rGO 2 wt%, and (f) β-Ga_2_O_3_-rGO 5 wt%. (g) UV-vis absorption spectra of the photocatalysts. (h) Tauc plot for the estimation of the bandgap of the photocatalysts; β-Ga_2_O_3_ (red line), β-Ga_2_O_3_-rGO 0.5 wt% (orange line), β-Ga_2_O_3_-rGO 1 wt% (yellow line), β-Ga_2_O_3_-rGO 2 wt% (light green line), and β-Ga_2_O_3_-rGO 5 wt% (green line). (i) The CO_2_ reduction mechanism of the proposed photocatalyst. The enlarged figure shows that the excited electrons in the photocatalyst are transferred to rGO and electrons are transferred to the C–OH group. It shows the three forms in which rGO is synthesized and shows the increase or decrease of the C–OH group according to the three cases.

The XPS survey spectra of the β-Ga_2_O_3_-based photocatalysts, as illustrated in Fig. 4a, reveal the surface elemental composition critical for understanding the photocatalytic activity. The high-resolution O 1s XPS core-level spectra of the pristine β-Ga_2_O_3_, represented in Fig. 4b, are meticulously deconvoluted into three well-defined peaks at binding energies of 530.8 eV, 531.5 eV, and 532.6 eV. These peaks correspond to different oxygen states within the material: lattice oxygen (O–Ga) signifying the oxygen bonded to gallium in the crystal structure, carbonyl oxygen (O=C) as a representative of oxygen in carbon–oxygen double bonds, and ether or alcohol oxygen (O–C) indicative of oxygen in carbon-oxygen single bonds.^82–86^ These states are crucial as they influence the electronic structure and chemical reactivity of the photocatalysts.

The emphasis on the O 1s peak analysis in this study is pivotal since the nature and reactivity of oxygen species play a significant role in photocatalytic processes. Oxygen species are actively involved in the generation and recombination of charge carriers, which are integral to the photocatalytic efficiency. In the β-Ga_2_O_3_-rGO 1 wt% sample, an additional peak emerges at approximately 533.6 eV in the O 1s spectrum (Fig. 4d), suggesting the presence of hydroxyl (C–OH) groups.^84^ This characteristic O–Ga bonding peak at 530.8 eV is a consistent feature across all β-Ga_2_O_3_-based samples, underlining its stability despite varying rGO doping levels. A prominent C–OH bonding peak, detected in Fig. 4d, is associated with the presence of hydroxyl groups on the rGO sheets, which are indicative of a specific interaction between the rGO and the photocatalyst.^8,87,88^

Fig. 4i illustrates a schematic of the photocatalytic mechanism, based on sample analysis. Light exposure activates electron–hole pair (EHP) generation in β-Ga_2_O_3_-rGO. These EHPs are preserved from recombination by their rapid transfer to the rGO layer, enhancing photocatalytic efficiency.^60,61^ The rGO acts as an electron scavenger, significantly improving the separation efficiency of photogenerated charge carriers. This enhanced separation is crucial, as it prevents the recombination of EHPs, allowing more electrons and holes to participate in photocatalytic reactions. Contaminants are adsorbed *via* OH groups on the catalyst surface^87,89–91^ and are reduced by the migrated electrons. The rGO's role in preventing EHP recombination and the presence of C–OH groups boost the photocatalyst's activity beyond β-Ga_2_O_3_ alone, emphasizing the importance of hydroxyl groups. Discontinuities or aggregations in the rGO sheet lead to more Epoxy (O–C) than hydroxyl (C–OH) groups, impacting photocatalytic efficiency.^92–95^ A schematic on Fig. 4i's right side explains C–OH bond variations in rGO- β-Ga_2_O_3_ composites. Fully-wrapped rGO retains its hydroxyl groups, whereas partially wrapped rGO leads to hydroxyl bonding with β-Ga_2_O_3_'s OH groups, reducing C–OH bonds.^96^ Over-stacked rGO forms denser layers, further decreasing C–OH bond presence.^97,98^

Hence, the observed increment in C–OH bonding in the β-Ga_2_O_3_-rGO 1 wt% sample suggests enhanced interfacial contact between β-Ga_2_O_3_ and rGO, which is conducive to improved charge separation – a key factor for photocatalytic efficiency. Furthermore, Raman analysis demonstrates that the original hydroxyl groups present in rGO are retained and stacked, while the structural defects resulting from encapsulation by β-Ga_2_O_3_ do not induce additional defects (Fig. S3†).^99,100^ Thus, the hydroxyl functionalities, which act as adsorption sites for contaminants, are preserved, potentially enhancing stronger interactions among pollutants, a crucial aspect in the photocatalytic degradation process.

The UV-vis absorption spectra of the β-Ga_2_O_3_-based photocatalysts with varying rGO content are presented in Fig. 4g, which highlights the materials' ability to absorb light across a broad spectrum ranging from the UV to the visible region (250–900 nm). Notably, the inclusion of rGO into the β-Ga_2_O_3_ matrix enhances visible-light absorption due to the π–π* transitions of the sp2-bonded carbon atoms in the rGO. This is evident from the absorption peak centered around 250 nm, attributed to the aromatic C=C bonds.

A shift in this peak's position with varying rGO content suggests alterations in the electronic structure of the composite, which can be correlated to changes in the conjugated system of the rGO.^101^

The determination of the optical band gap through tauc plots is a vital step in understanding the electronic properties of the photocatalysts.^102^ As shown in Fig. 4h, the results indicate a decrease in the band gap as the rGO content increases, revealing a tunability of the material's electronic structure *via* rGO integration. This tuning of the band gap is significant as it implies an increased range of photon energies that the material can utilize for photocatalytic reactions. Starting with pristine Ga_2_O_3_ at 4.35 eV, the band gap narrows significantly to 3.02 eV with the addition of rGO, and further incorporation of rGO reduces it to 2.74 eV. This reduction in band gap is indicative of the potential for enhanced photocatalytic activity under visible light, which is particularly relevant for applications in environmental remediation and solar energy conversion. Given that the photocatalysts exhibit diverse morphologies and optoelectronic properties with different rGO loadings, it becomes imperative to investigate their photocatalytic activities in a dynamic setting. Therefore, real-time spectroscopic analysis will be employed to study the degradation of pollution under irradiation, enabling a direct comparison of the photocatalytic efficiencies of these rGO-modified catalysts.

### 2.2 Real-time detection of photocatalysis mechanisms for β-Ga_2_O_3_-rGO samples

**Fig. 5:**
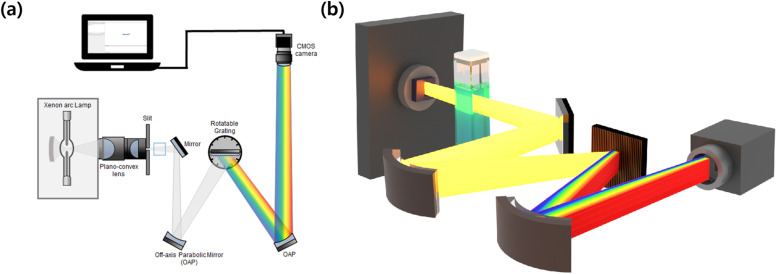
(a) 2D and (b) 3D schematic images of real-time absorbance spectroscopy. The setup utilizes a broadband “white” light focused into a 100 μm vertical slit aperture *via* two plano-convex lenses. A light beam passing through the sample is collimated by an off-axis parabolic mirror (OAP) and is scattered upon encountering the grating. The scattering spectrum from the grating is recollimated by the second OAP mirror. The scattering spectrum from the grating is recorded by a CMOS camera.

To evaluate the effect of rGO content on the enhancement of photocatalytic performance, real-time spectroscopic analysis was conducted on these photocatalysts, each containing varying quantities of rGO (0 wt%, 0.5 wt%, 1 wt%, 2 wt%, and 5 wt%). Fig. 5 depicts a schematic diagram of a simultaneous spectroscopy used to detect the decomposition of an MB aqueous solution in real time with β-Ga_2_O_3_ based photocatalysts, including the β-Ga_2_O_3_-rGO composites. The main concept of this setup is to employ a complementary metal-oxide-semiconductor (CMOS) camera that detects a broad spectrum of a broadband light source transmitted through a sample and distributed by a grating.^103^ Unlike a traditional commercial UV-vis spectroscopy, which adjust the grating to detect only one color at a time, this optical configuration captures all spectral information in real-time from the UV to Visible range without wavelength tuning.

In this setup, a broadband Xenon arc source is first focused onto the entrance vertical slit aperture using two plano-convex lenses. The focused light that passes through the slit is transmitted through the sample cuvette and then collimated with an off-axis parabolic mirror (OAP). The collimated light is then dispersed by a grating, and the resulting spectrum is collimated once again with another OAP. Ultimately, this dispersed spectrum is recorded by a CMOS camera, enabling the acquisition of the sample's real-time absorption spectrum.

**Fig. 6:**
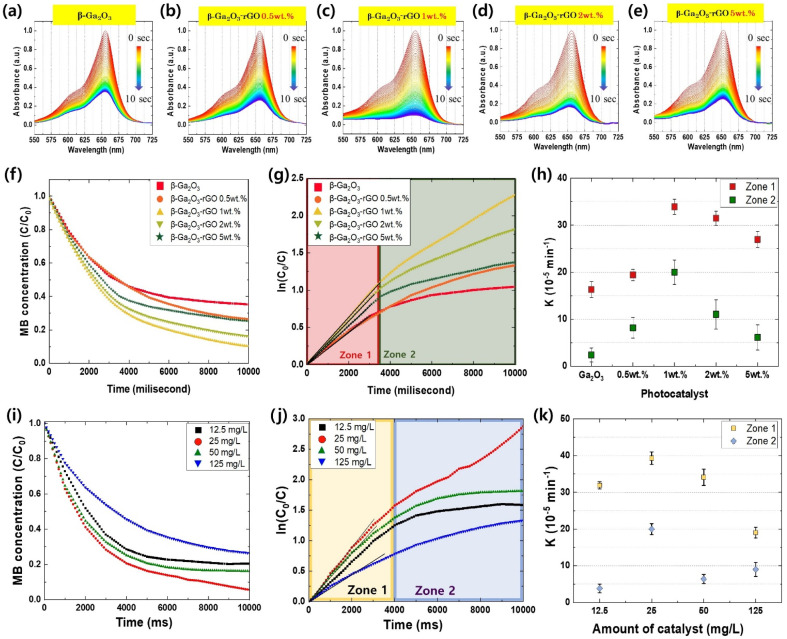
(a–e) MB photolysis spectrum for the β-Ga_2_O_3_-rGO photocatalyst at different rGO concentrations: (a) β-Ga_2_O_3_, (b) β-Ga_2_O_3_-rGO 0.5 wt%, (c) β-Ga_2_O_3_-rGO 1 wt%, (d) β-Ga_2_O_3_-rGO 2 wt%, and (e) β-Ga_2_O_3_-rGO 5 wt%. (f) The decomposition rate of MB concentration per hour (*C*/*C*_0_) at the MB peak and (g) the natural logarithm of the initial to actual concentration ratio (ln(*C*_0_/*C*)) per hour for β-Ga_2_O_3_-rGO photocatalysts. (h) Reaction rate constant K for β- Ga_2_O_3_-rGO photocatalysts. (i) The decomposition rate of MB concentration per hour (*C*/*C*_0_) at the MB peak and (j) the natural logarithm of the initial to actual concentration ratio (ln(*C*_0_/*C*)) per hour for β-Ga_2_O_3_-rGO 1 wt% photocatalyst at four different dosages (12.5 mg L^−1^, 25 mg L^−1^, 50 mg L^−1^, and 125 mg L^−1^). (k) Reaction rate constant *K* for the β-Ga_2_O_3_-rGO 1 wt% photocatalysts samples at varying dosages.

The photodegradation of methylene blue (MB) was monitored in real-time as shown in Fig. 6a–e. The pristine β-Ga_2_O_3_ exhibits lesser photocatalytic performance relative to the enhanced activity of β-Ga_2_O_3_ when integrated with rGO. Through FE-SEM and TEM images, we have confirmed the association between the quantity of rGO and morphology. Furthermore, it was observed that the photodegradation efficiency was saturated and subsequently decreased due to this morphology. A noticeable escalation in photodegradation is observed within a span of 10 seconds when the concentration of rGO added to β-Ga_2_O_3_ is increased, peaking at 1 wt% rGO. Beyond this point, further additions of rGO result in a decline in photodegradation. The incorporation of precisely 1 wt% rGO leads to the total bleaching of MB within just 10 seconds, signifying the pinnacle of photocatalytic efficiency. The presence of rGO facilitated electron–hole transport, preventing recombination and enabling more effective oxidation and reduction of MB. Fig. 6f showed that the photodegradation rates (*C*/*C*_0_) of MB increased with increasing rGO content, peaking at 90.4% for the 1 wt% rGO sample. However, a further increase in rGO content led to a decrease in photodegradation efficiency due to the formation of flake-like rGO structures, interfering with energy transfer and resulting in a decreased MB decomposition rate of 75.7% for the 5 wt% rGO sample. Comparatively, the pure β-Ga_2_O_3_ photocatalyst exhibited a degradation performance approximately 10% lower than the synthesized photocatalyst, attributed to limited electron–hole pair maintenance and transport capabilities.

The efficiency of the photocatalysts was assessed by determining the reaction rate constants (*K*), derived from the slopes of the ln(*C*_o_/*C*) plots for each sample.^104–107^ These plots, as displayed in Fig. 6g and h, divulge two distinctive zones, each characterized by different rate constants. These variations in the rate constant, *K*, can be attributed to the morphology of β-Ga_2_O_3_-rGO and the rGO content. During the early stage of the photodegradation process, referred to as Zone 1, the rate constant *K* bifurcates into two categories: samples devoid of rGO encapsulation (β-Ga_2_O_3_ and β-Ga_2_O_3_-rGO 0.5 wt%), and those encapsulated with rGO (rGO 1 wt%, 2 wt%, and 5 wt%). The K values for the rGO encapsulated samples are approximately 1.5 to 2 times greater than their non-rGO encapsulated counterparts. This pattern indicates that the encapsulation of β-Ga_2_O_3_ with rGO is primarily responsible for determining the rate constant, and thereby the photocatalytic efficiency during the initial phase of photodegradation. This likely arises from more efficient preservation and transfer of EHPs facilitated by the encapsulation process.

In contrast, during the latter stage of photodegradation, or Zone 2, the differences between the samples become less distinct, except for the 1 wt% rGO sample, where β-Ga_2_O_3_ is effectively encapsulated by rGO. This finding aligns with the structural interferences illustrated in Fig. 2 and 3. Consequently, while rGO boosts photocatalytic performance to a certain extent, an excess can create structural obstacles that obstruct energy transfer, thereby diminishing the overall efficiency of photodegradation. In essence, judicious incorporation and management of rGO content is pivotal for enhancing the efficiency of β-Ga_2_O_3_-based photocatalysts. While rGO considerably enhances photocatalysis by facilitating electron–hole transport and preventing recombination, it's crucial to balance its content to evade any potential detrimental effects on the performance of photodegradation.

The earlier sections of our study underscored the substantial impact of the rGO synthesis form on photocatalyst performance. To further examine the underlying mechanisms that dictate the effectiveness of the photocatalyst, we conducted a series of dosage experiments. Optimizing dosage is a critical aspect of any experimental investigation, as it can significantly influence the performance outcomes, allowing us to achieve the highest photocatalytic performance while conserving resources. To identify the optimal dosage of the β-Ga_2_O_3_-rGO 1 wt% sample for photocatalysis, we prepared four different dosages. These were derived from the most effective photocatalysis results gleaned from our prior experiments and are outlined in detail in Table 2.

Fig. 6i visually represents the extent of MB photodegradation at 100 millisecond intervals for these four dosages: 12.5 mg L^−1^, 25 mg L^−1^, 50 mg L^−1^, and 125 mg L^−1^. This comparison of photodegradation based on different dose suggests the existence of an optimized photocatalyst ratio and indicates that when a larger quantity of catalyst is introduced than this ratio, photodegradation efficiency decreases. The dosage of 25 mg L^−1^ stood out, demonstrating the most superior photocatalytic activity among the tested dosages. The degrees of photocatalysis achieved were 76.4%, 87.5%, 81.4%, and 64.3% for the 12.5 mg L^−1^, 25 mg L^−1^, 50 mg L^−1^, and 125 mg L^−1^ dosages, respectively. As such, this suggests that 25 mg L^−1^ is the optimal dosage to maximize the photocatalytic activity of the β-Ga_2_O_3_-rGO 1 wt% sample. The results suggest a diminishing return of photocatalysis efficiency with increasing photocatalyst dosage, which can be attributed to a decrease in the available surface area for reaction. Notably, a significant drop in photocatalytic efficiency was observed when the amount of photocatalyst was increased to 125 mg L^−1^. There are several factors that contribute to the diminished photodegradation in the presence of excess photocatalysts. Firstly, the filter effect: an increased amount of photocatalyst can introduce inert particles into the solution, blocking light transmission and disrupting the reaction.^108^ Secondly, a decrease in the surface area: an excessive amount of photocatalysts can aggregate into clusters, reducing the available surface area for reaction.^109^ Finally, a decrease in excited energy: collisions between light-excited and ground-state catalyst particles can lead to a ground-state scenario, thereby reducing the reaction efficiency.^110^

**Fig. 7:**
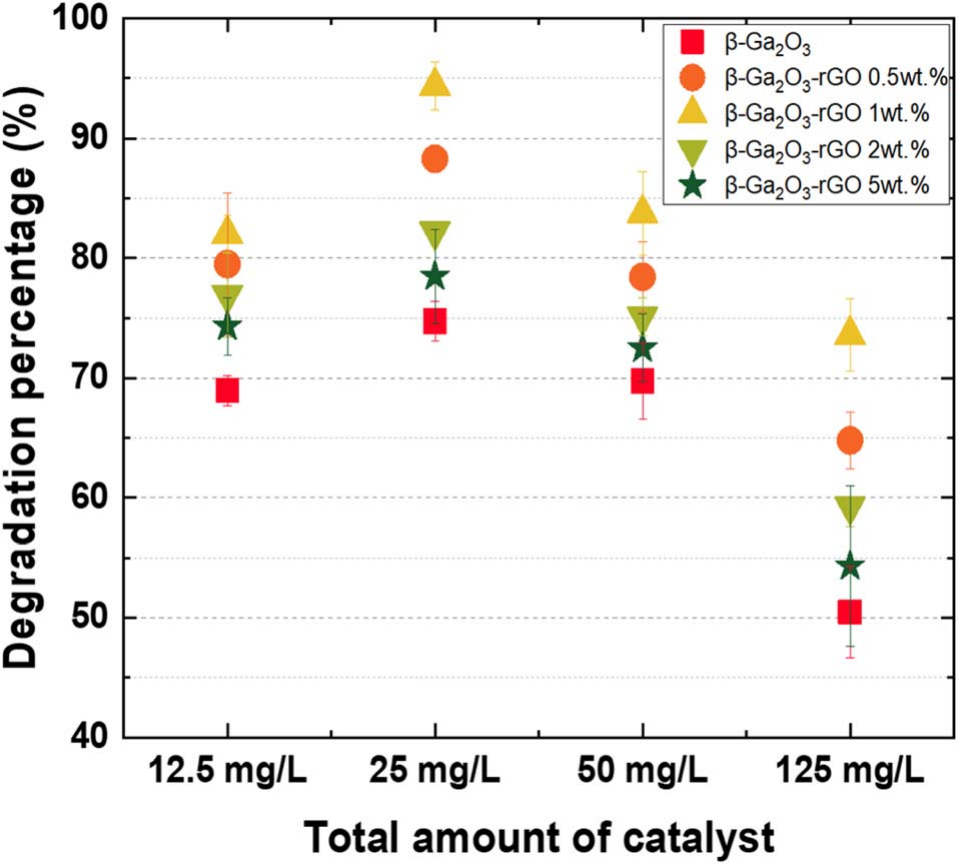
Photocatalytic degradation percentages of MB by various photocatalysts; β-Ga_2_O_3_ (red square), β-Ga_2_O_3_-rGO 0.5 wt% (orange circle), β-Ga_2_O_3_-rGO 1 wt% (yellow upward triangle), β-Ga_2_O_3_-rGO 2 wt% (light green downward triangle), and β-Ga_2_O_3_-rGO 5 wt% (green star) at varying dosages (12.5 mg L^−1^, 25 mg L^−1^, 50 mg L^−1^, and 125 mg L^−1^).

To investigate the photodegradation mechanisms, we turned to the ln(*C*_0_/*C*) plot and computed the reaction rate constant, as displayed in Fig. 6j and k, respectively. During Zone 1, the graph profiles for most samples were notably similar, with the exception of the sample loaded with an excessive amount of catalyst (125 mg L^−1^). In contrast, Zone 2 showed samples with an optimal catalyst loading (25 mg L^−1^) demonstrating a significantly higher *K* value compared to those with either insufficient or excessive amounts of catalyst. This occurrence can be ascribed to the increased quantity of inactivated photocatalyst, which ironically results in a reduced efficiency due to the aforementioned factors (Fig. 7).

### 2.3 Detection of CO_2_ reduction

**Fig. 8:**
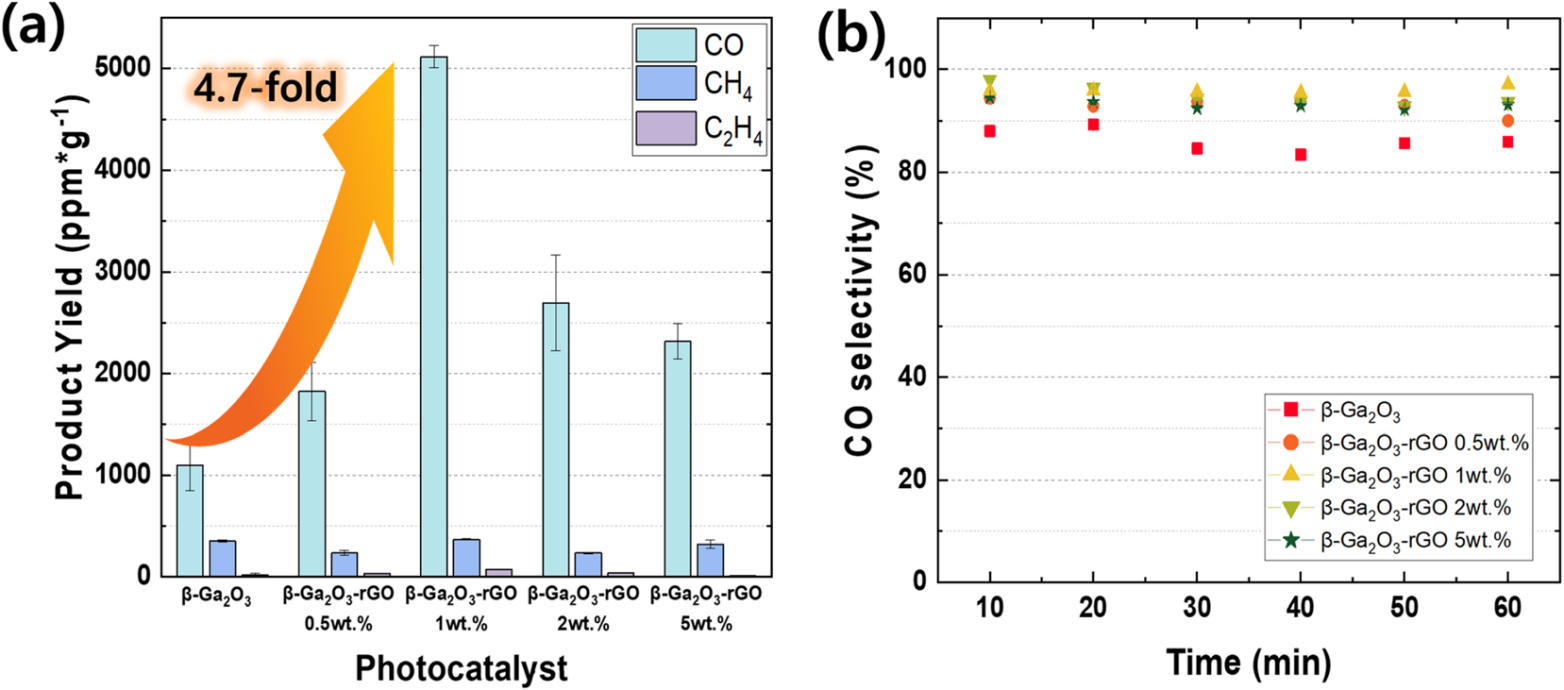
(a) Cumulative production of CO, CH_4_, and C_2_H_4_ over a 60 minute experimental duration using β-Ga_2_O_3_-based photocatalysts (pristine β-Ga_2_O_3_ and β-Ga_2_O_3_-reduced graphene oxide (rGO) catalysts). The total product yields for CO, CH_4_, and C_2_H_4_ were quantified using gas chromatography, which sampled the gases every 10 minutes for 1 minute, resulting in six measurements throughout an hour. (b) Selectivity towards CO production by the five β-Ga_2_O_3_-based photocatalysts across various irradiation times.

The performance of this photocatalyst exhibits distinct advantages, particularly in the context of CO_2_, as elucidated in the experimental section. The process involves the conversion of CO_2_ gas, adsorbed onto the catalyst surface, into CO, CH_4_, and C_2_H_4_, as shown in Fig. 8a, where gas chromatography reveals the yields of each product.^111^ The total product yields for CO, CH_4_, and C_2_H_4_ were quantified using gas chromatography, which sampled the gases every 10 minutes for 1 minute, resulting in six measurements throughout an hour. Incorporating 1 wt% rGO into the β-Ga_2_O_3_ photocatalyst composition markedly enhances CO production, achieving an optimal yield of 5116.4 ppm g^−1^. This yield is approximately 4.7 times higher than that of the pristine β-Ga_2_O_3_. Such a significant increase indicates a peak in photocatalytic activity at this specific rGO concentration, distinguishing it from the yields obtained with other rGO weight percentages. The data suggest an optimal rGO loading for maximizing photocatalytic performance. The yields for different samples were observed as follows: 1100.3 ppm g^−1^ for pristine β-Ga_2_O_3_, increasing to 1821.6 ppm g^−1^ with 0.5 wt% rGO, and peaking at 1 wt% rGO. Higher concentrations of rGO, such as 2 wt% and 5 wt%, did not proportionally increase the CO yield (2698.5 ppm g^−1^ for β-Ga_2_O_3_-rGO 2 wt% and 2306.7 ppm g^−1^ for β-Ga_2_O_3_-rGO 5 wt%), which implies that an excess of rGO beyond the optimal point does not translate to further catalytic benefits. The stability and selectivity of the photocatalytic process for the β-Ga_2_O_3_-rGO 1 wt% sample is further evidenced by the CO selectivity rates, which consistently exceed 98% over time, as illustrated in Fig. 8b. This represents a significant stride in selectivity when compared to the pristine β-Ga_2_O_3_. Given the minimal production of ethylene, supplementary measurements were undertaken using a highly sensitive ethylene detector to conduct a comparative analysis of each catalyst (Fig. S5†). Within this investigation, porous nanorod β-Ga_2_O_3_ significant increase in C_2_H_4_ production compared to the commercially available nanoparticle TiO_2_. These research findings highlight the promising functionality of β-Ga_2_O_3_-rGO photocatalysts for practical environmental applications, providing a notably efficient and selective option for CO_2_ photoreduction.

## 3. Conclusion

In summary, this investigation introduces a novel β-Ga_2_O_3_-rGO photocatalyst that demonstrates substantial improvements in photocatalytic performance. The synergy between β-Ga_2_O_3_ nanorods and rGO nanosheets, as revealed by SEM and HR-TEM, creates an architecture that is highly conducive to catalytic efficiency. XPS analyses have further elucidated the role of rGO functional groups, such as C–OH, in promoting pollutant adsorption and enhancing charge separation. This structural innovation, coupled with the altered light absorption properties detected by UV-vis spectroscopy, marks a significant stride in photocatalyst design.

The β-Ga_2_O_3_-rGO 1 wt% sample emerged as distinctly superior in real-time spectroscopy assessments of dye degradation rates, outperforming other rGO concentrations. This optimized rGO addition significantly reduces electron–hole recombination, thereby increasing the efficiency of oxidation–reduction reactions crucial for pollutant degradation. The research identifies 25 mg L^−1^ as the optimal catalyst concentration for peak photocatalytic performance, with higher concentrations proving less effective.

The study presents a significant advancement in photocatalysis, showcasing the novel β-Ga_2_O_3_-rGO 1 wt% sample's remarkable efficiency in CO_2_ reduction. Achieving an impressive increase in CO product yield and 98% CO selectivity, this catalyst is distinguished from others in the manufactured rGO content, highlighting its potential as a superior choice for CO_2_ conversion applications. The β-Ga_2_O_3_-rGO 1 wt% catalyst, therefore, stands as a significant advancement in the field, with promising implications for environmental remediation. The findings not only showcase the catalyst's efficacy but also the utility of real-time spectroscopy as a tool for optimizing and advancing photocatalytic material development.

## 4. Material and methods

### 4.1 Sample preparation

To improve photocatalytic activity, research is underway to develop photocatalysts with smaller particle sizes and porous structures (Fig. S7†).^8,112^ In this study, we aimed to synthesize Ga_2_O_3_ photocatalysts with a nanorod structure by adding 10 g of Ga(NO_3_)_3_ powder to 50 mL of deionized water (DI water) and 15 mL of ammonium hydroxide, since hydrothermal synthesis at pH 5 promotes the growth of crystals in the form of nanorods formation.^113,114^ The mixed solution was stirred at 60 °C for 2 hours, transferred to a Teflon container, placed in a sealed autoclave, and held at 150 °C for 5 hours, after which it was dried at room temperature for 24 hours. After synthesis, the precursor (GaOOH) underwent sintering at 900 °C for 5 hours, leading to the formation of β-Ga_2_O_3_ and the elimination of OH groups, ultimately yielding porous nanorods of β-Ga_2_O_3_.^115^

To improve the photodegradation efficiency, β-Ga_2_O_3_ in the form of porous nanorods is combined with reduced graphene oxide (rGO) as a photocatalyst.^72–74,77^ Initially, pure GO powder is prepared by the Hummers' synthesis method.^116^ To prepare the β-Ga_2_O_3_-rGO photocatalyst, sample groups were prepared with four different doses of rGO as described in Table 1. Each combination in the table was placed in a Teflon container containing 50 mL of DI water and stored in a sealed autoclave at 150 °C for 5 hours. The synthesized solution was then dried at room temperature for 24 hours.

**Table 1:** 4 different β-Ga_2_O_3_-rGO nanoparticle concentration

Sample	β-Ga_2_O_3_ catalyst (mg)	GO powder (mg)	rGO (wt%)
β-Ga_2_O_3_	400	0	0
β-Ga_2_O_3_-rGO 0.5 wt%	398	2	0.5
β-Ga_2_O_3_-rGO 1 wt%	396	4	1
β-Ga_2_O_3_-rGO 2 wt%	392	8	2
β-Ga_2_O_3_-rGO 5 wt%	380	20	5

We conducted experiments on the composition and capacity of the photocatalyst. Firstly, the experiments based on composition were evaluated by conducting MB photocatalytic degradation using a photocatalyst dosage of 1.5 mg, as synthesized according to the criteria shown in Table 1. Secondly, experiments on dosage were performed by preparing four samples with different photocatalyst doses as shown in Table 2 to investigate the effect of photocatalyst dosage on photodecomposition. A reference solution without MB was also prepared as described in Table 2. A rapid initial decrease in dye concentration occurs due to the adsorption of the dye onto the catalyst. To eliminate such artifacts, this experiment incorporates a stabilization period. The sample solutions were shielded from external light with aluminum foil to prevent unintended photodegradation and stirred for 30 minutes to ensure complete adsorption.

**Table 2:** Four different MB samples with β-Ga_2_O_3_-rGO 1 wt% nanoparticle concentration

Sample	Ethanol (ml)	Methylene blue (mg)	β-Ga_2_O_3_-rGO (mg)	Weight of photocatalyst to total weight (wt%)
12.5 mg L^−1^	40	0.2	0.5	1
25 mg L^−1^	40	0.2	1	2
50 mg L^−1^	40	0.2	2	5
125 mg L^−1^	40	0.2	5	11

### 4.2 Characterization

#### 4.2.1 Material analysis

The crystal structure and phase of β-Ga_2_O_3_, as well as its comparison with GaOOH, were analyzed using X-ray diffraction (XRD, Ultima IV, Rigaku) with Cu K-alpha radiation. To capture the diagrams, we employed a standard incident angle of 0.5. The morphologies of β-Ga_2_O_3_ and the synthesized β-Ga_2_O_3_-rGO composite were characterized using Field Emission-Scanning Electron Microscopy (FE-SEM, JSM-7610F-Plus, JEOL) equipped with energy dispersive X-ray spectroscopy (EDS) at an accelerator voltage of 20 kV. The UV-vis spectrophotometer (V650, Jasco) equipped with an integrating sphere attachment for diffuse reflectance analysis was employed to examine the reflectance spectra and subsequently determine the optical band gap by plotting the Tauc plot of the Kubelka–Munk function based on absorbance data. In preparing the cross-sectional TEM sample, we utilized the Dual-Beam focused ion beam (FIB) (Scios2, Thermo Fisher Scientific) with a 30 kV gallium (Ga) ion beam for the lift-out technique. Subsequently, the sample was investigated by a high-resolution (HR) transmission electron microscope (TEM) (Talos F200X, Thermo Fisher Scientific) attached to an EDS operating at an acceleration voltage of 200 kV. The surface chemical states of the samples were characterized by X-ray photoelectron spectroscopy (XPS, Theta Probe AR-XPS system, Thermo Fischer Scientific) using a monochromatic Al Kα X-ray source (1486.6 eV). Binding energies were calibrated with respect to the C 1s peak at 285.0 eV. The Raman spectra were acquired with a laser Raman microscope (LabRAM Soleil, Horiba Jobin Yvon) using a 532 nm excitation laser.

#### 4.2.2 Detection of CO_2_ reduction

CO_2_ gas was supplied continuously *via* a mass flow controller to maintain a consistent flow rate. This gas flowed through a silicon pipe, sequentially passing through a water bubbler and then into a gas flow cuvette. Within the cuvette, a photocatalyst facilitated the reduction of CO_2_ to CO, CH_4_, and C_2_H_4_, a process aided by a 15 W UVC lamp with a 254 nm wavelength. The composition of the resulting reduced gas mixture was then meticulously monitored at 10 minute intervals using gas chromatography.

## Data availability

The data supporting this article have been included as part of the ESI.†

## Author contributions

Hyein Jung: conceptualization, methodology, validation, formal analysis, investigation, data curation, writing – original draft, writing – review & editing, visualization. Hangyeol Choi: validation, formal analysis, investigation, data curation. Yu-Jin Song: formal analysis, investigation. Jung Han Kim: validation, formal analysis, investigation. Yohan Yoon: conceptualization, methodology, resources, supervision, funding acquisition, validation, formal analysis, investigation, data curation, writing – review & editing, visualization, project administration.

## Conflicts of interest

The authors declare no conflict of interest.

## Supplementary Material

NA-006-D4NA00408F-s001
