# Supplementary material for: Synergistic augmentation and fundamental mechanistic exploration of β-Ga2O3-rGO photocatalyst for efficient CO2 reduction
Source: Nanoscale Adv. 2024 Jul 15;6(18):4611–24. doi: 10.1039/d4na00408f (PMC11385812; doi:10.1039/d4na00408f)
Supplement: NA-006-D4NA00408F-s001 [file NA-006-D4NA00408F-s001.pdf]

Electronic Supplementary Material (ESI) for Nanoscale Advances.
This journal is © The Royal Society of Chemistry 2024

Synergistic Augmentation and Fundamental Mechanistic Exploration of β-Ga_2O_3-rGO Photocatalyst for Efficient CO_2 Reduction

Hye-In Jung[1], Hangyeol Choi[1], Yu-Jin Song[2], Jung Han Kim[2], and Yohan Yoon[1,]*

[1] Korea Aerospace University, Department of Materials Engineering, Goyang, Republic of Korea

[2] Dong-A University, Department of Materials Science and Engineering, Busan, Republic of Korea

[3] School of Materials Science & Engineering, Kyungpook National University, Daegu, Republic of Korea

The mechanism behind the formation of microstructures originates from the sample fabrication process. During synthesis, the capillary action leads rGO sheets to adhere differently from β-Ga_2O_3 [1]. As the quantity of rGO increases, the sheets also expand, eventually enveloped the β-Ga_2O_3. However, energy accumulates at the unbound edges of the rGO sheets in this state [2, 3], prompting additional rGO sheets to stack at these edges to reduce the energy. This series of actions results in an increased surface area of rGO enveloping the β-Ga_2O_3, leading to a phenomenon where, with rising rGO content, instead of encapsulating the surface area, it conglomerates. Another study proposed that, in thin sheets, an increase in thickness leads to a stable form where conglomerating at the edges or within specific regions, rather than enveloping the surface, is more stable [4, 5]. As shown in Fig. S1, as the rGO content increases and the thickness escalates, energy concentrates at the edges of the rGO sheets, creating a folded appearance due to stacking at those regions. Subsequently, more rGO is layered onto these areas, forming the shape of an rGO flake.

We examined the sample's morphology using FE-SEM and investigated the sample's interfaces via TEM, as presented in Fig. S2, offering cross-sectional images into the nano-scale architecture of the β-Ga_2O_3-rGO 2 wt.% sample. Fig. S1b represents an enlargement of the red region shown in Fig. S1a. TEM images clearly reveal the interfaces between β-Ga_2O_3 and rGO. When compared to Fig. 3a (β-Ga_2O_3-rGO 1 wt.%), it is evident that the rGO layer encapsulates β-Ga_2O_3 more extensively, with a significantly greater thickness (around 55 nm).

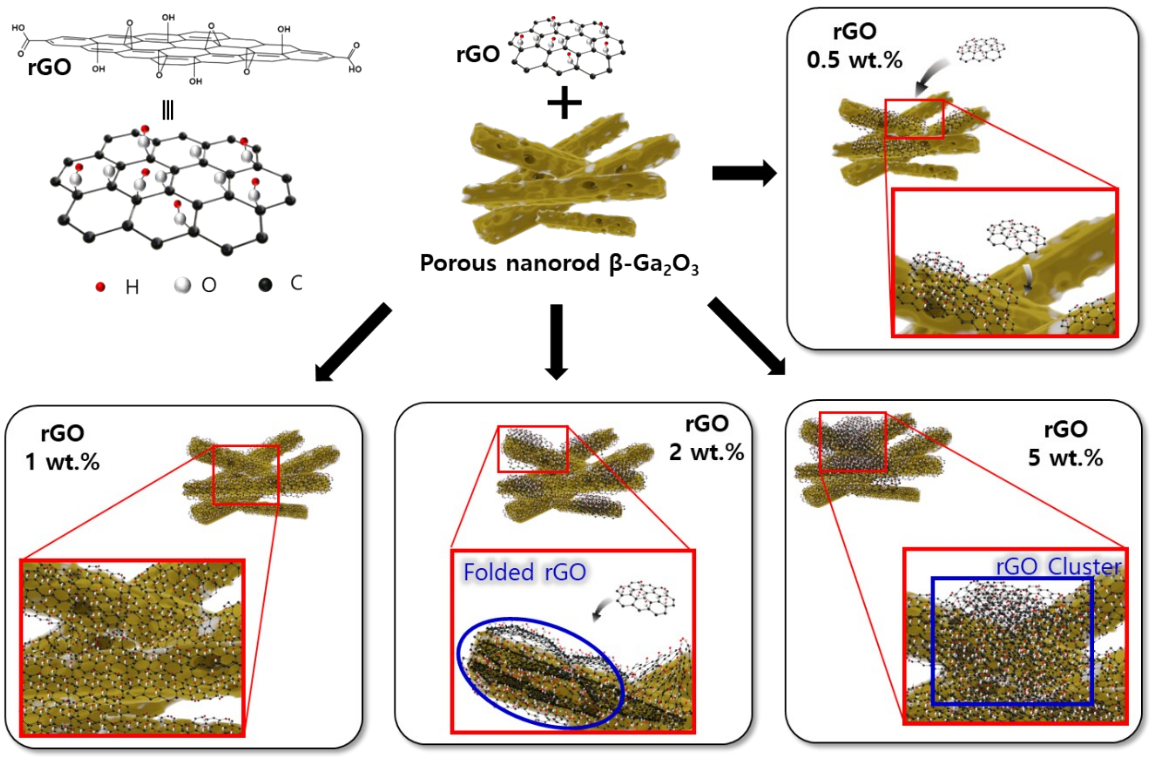

Fig. S1 Illustration of β-Ga_2O_3-rGO composite formation with varying rGO concentrations. At 0.5 wt.%, rGO sheets begin adhering to the porous β-Ga_2O_3 nanorods. With an increase to 1 wt.%, the rGO sheets cover more surface area, creating a more uniform encapsulation. At 2 wt.% and beyond, the rGO sheets exhibit increased stacking and wrinkling at the edges, leading to the formation of rGO clusters at 5 wt.%, which indicates a transition from encapsulation to agglomeration. This microstructural evolution reflects the balance between capillary action during synthesis and energy minimization at the rGO edges, influencing the photocatalytic efficiency and stability of the composite.

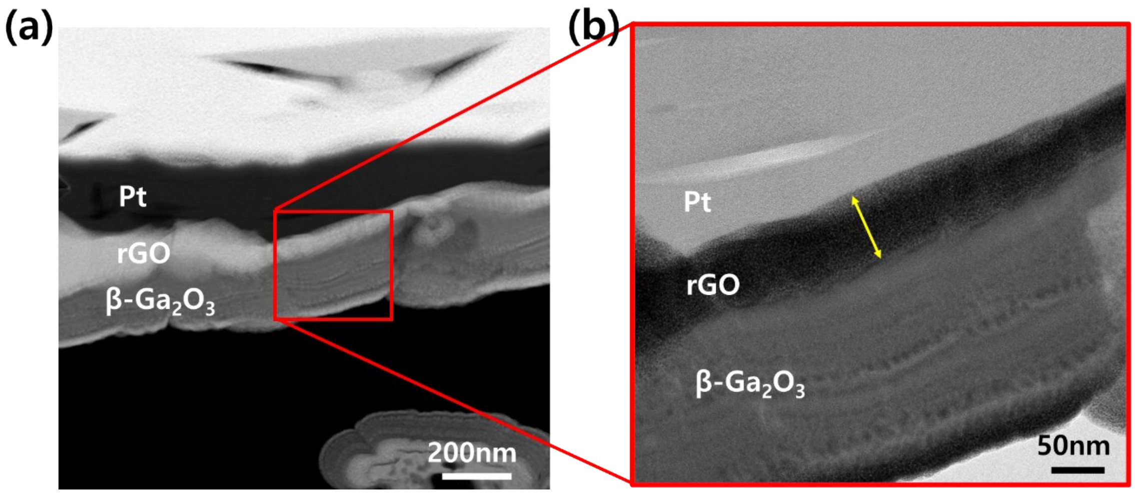

Fig. S2 TEM image of the β-Ga_2O_3-rGO 2wt.% photocatalyst; cross-section image (a), an image in which the red square area is enlarged from the corresponding image (b).

Raman studies of β-Ga_2O_3-rGO samples

As seen in Fig. S3, the Raman spectrum of rGO predominantly exhibits the D-band peak(1353cm^{-1}) attributed to sp3 defects and the G-band peak(1597cm^{-1}) originating from in-plane vibrations of sp2 carbon atoms. No other peaks are observed, indicating the absence of impurities in the rGO sample[6].

In the Raman spectra of β-Ga_2O_3-rGO 0.5wt.%, both the characteristic peaks of β-Ga_2O_3 and rGO are observed concurrently. As the rGO content increases, the D and G bands become more prominent, while the peaks associated with β-Ga_2O_3 notably diminish. This phenomenon arises from the inherent differences in scattering intensity between rGO and β-Ga_2O_3. Notably, at the transition point of β-Ga_2O_3-rGO 1wt.%, the I_D/I_G ratio experiences a slight increase from 0.92 to 1.00. The increase in the I_D/I_G ratio is attributed to structural disorder[7, 8]. As confirmed by FESEM and TEM, at 1 wt.%, the catalyst encapsulation differs from previous concentrations, leading to a significant increase in photodegradation efficiency. Therefore, this indicates that the rGO layers initially undergo simple stacking but then encapsulate the catalyst upon reaching a critical concentration. Structural defects occur in the rGO layers to facilitate the encapsulation of the catalyst. The rGO layers are then continuously stacked with the catalyst encapsulated, resulting in a fixed I_D/I_G ratio of 1.00 as only the thickness increases.

These findings reaffirm that β-Ga_2O_3-rGO 1wt.% represents the critical concentration. Except for the slight increase in the I_D/I_G ratio from 0.92 to 1.00, no significant changes in peak width or peak shift are observed, suggesting that β-Ga_2O_3 was synthesized with rGO without significant defects.

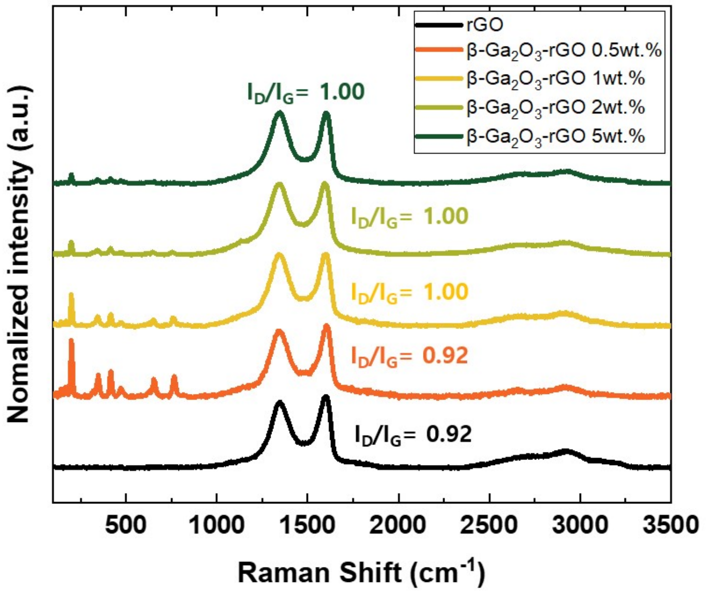

Fig. S3 Raman spectra of rGO and β-Ga_2O_3-rGO photocatalysts.

Real-time detection of photocatalysis mechanisms for β-Ga_2O_3-rGO samples

Figures S4a and S4b depict contour maps that translate an image, recorded by a CMOS camera, into a broadband spectrum with the wavelength plotted along the x-axis and pixels on the y-axis. This conversion is facilitated through wavelength calibration [9]. Fig. S4a presents the light spectrum transmitted through the reference solution, as discussed in the experimental stage. On the other hand, Fig. S4b represents the light spectrum that has passed through the MB-containing sample. The spectrum of MB is then derived by subtracting the signal values of these two images, as shown in Fig. S4c [9]. Fig. S4d illustrates the absorption spectra for the MB specimen, taken from three distinct dash-lined paths within the absorption contour map (as shown in Fig. S4c). Crucially, the spatial characteristics of the samples are represented along the y-axis (camera pixel), allowing any spatial variations in the illuminated zones of the samples to be monitored.

Figure S5a indicates a slight delay of approximately 50 seconds in ethylene (C_2H_4) production when activated by UV light. The ethylene yields for the series of catalysts—pristine β-Ga_2O_3, and those with 0.5 wt.%, 1 wt.%, 2 wt.%, and 5 wt.% rGO—were measured at 16.3 ppm·g^{-1}, 50.5 ppm·g^{-1}, 58.8 ppm·g^{-1}, 32.1 ppm·g^{-1}, and 23.5 ppm·g^{-1}, respectively. These are in line with the enhanced photocatalytic efficiency observed in gas chromatography analyses, which indicated improved performance with the introduction of rGO. The pristine β-Ga_2O_3, with its porous nanorod structure, achieved 1.3 times higher C_2H_4 production than that of the commercially available nanoparticle TiO_2. Remarkably, the 1 wt.% β-Ga_2O_3-rGO sample demonstrated a significant 4.8 times increase in C_2H_4 yield, underscoring its superior photocatalytic activity.

Figure S5b outlines the correlation between C_2H_4 product yield and methylene blue (MB) degradation percentage for the various photocatalysts. The data reveal that at the optimal concentration of 25mg/L, the peak in MB degradation percentage coincides with similar trends in the C_2H_4 product yield for all five photocatalysts, regardless of rGO content. This observation suggests a shared activation mechanism between CO_2 photoreduction and dye degradation in an aqueous solution, highlighting a parallelism in the photocatalytic processes.

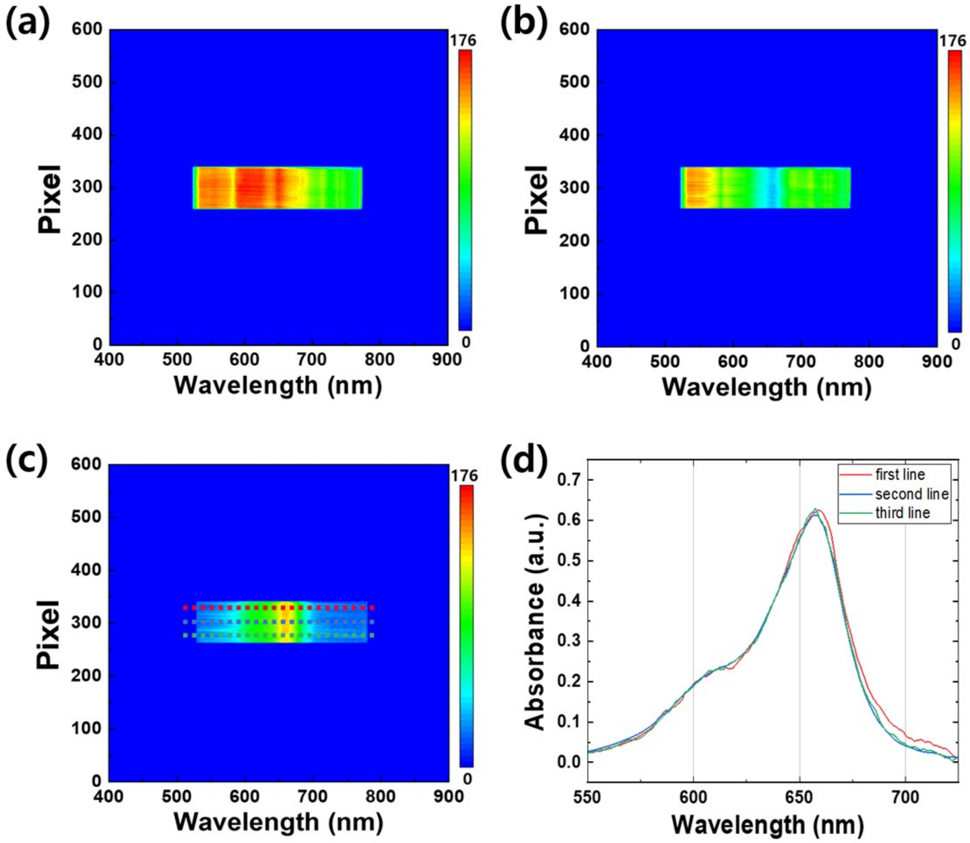

Fig. S4 Representative contour images obtained by pixel-wavelength conversion of the spectrum taken by a camera when the light passes through (a) the reference solution, (b) the MB solutions. (c) A representative absorbance contour map of the MB sample obtained by subtracting the spectra

of the two solutions. (d) Three absorbance spectra of the MB solutions of three different areas in c.

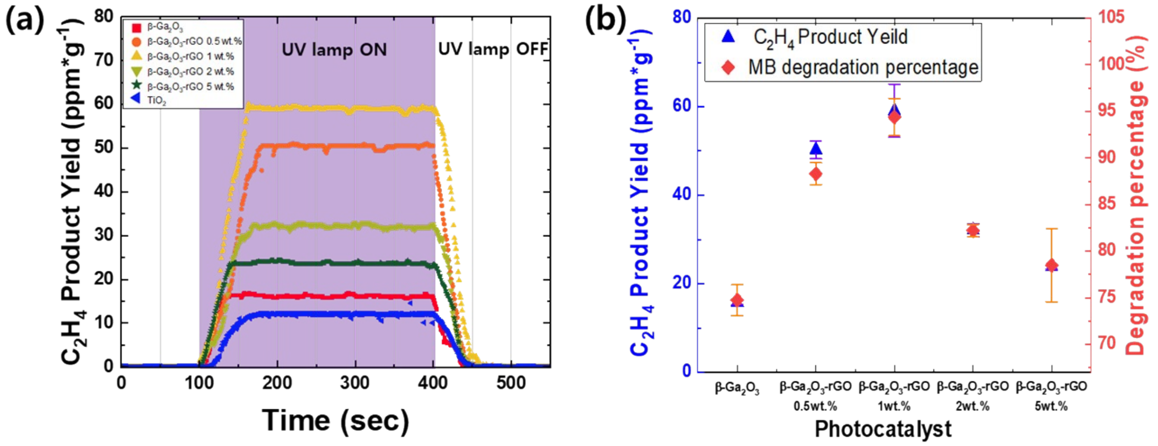

Fig. S5 (a) C_2H_4 production via β-Ga_2O_3-based photocatalysts (β-Ga_2O_3, β-Ga_2O_3-rGO 0.5wt%, β-Ga_2O_3-rGO 1wt.%, β-Ga_2O_3-rGO 2wt.%, and β-Ga_2O_3-rGO 5wt.%) and TiO_2. The graph illustrates an upward trend in C_2H_4 production observed from 100 to 400 seconds under UV lamp exposure. (b) C_2H_4 product yield and the MB degradation percentage achieved by various photocatalysts. For MB degradation percentages, the dosage of the samples was 25mg/L.

Comparison of the photodegradation efficiency of methylene blue on various photocatalysts

Compared to photocatalysts currently under investigation, our engineered photocatalyst exhibits superior dye degradation performance. Various photocatalysts, including TiO_2, are being explored for dye removal applications [10-19]. While xenon lamps with a broad spectrum of wavelengths, akin to natural light, are commonly utilized, ultraviolet light sources are also employed to enhance efficiency in Ga_2O_3 and other photocatalysts due to their large bandgap. In this study, we achieved an outstanding photocatalytic efficiency of 94.8% using a broad-spectrum light source. This exceptional performance can be primarily attributed to the reduced recombination of EHPs facilitated by the separation and transport of these pairs. What further distinguishes our results apart is the rapid degradation achieved through rGO synthesis. In contrast, other photocatalysts have demonstrated over 90% degradation efficiency, with an average time requirement of 120 minutes, and the shortest reported time for dye degradation being 15 minutes. Conversely, our photocatalyst achieved a remarkable 94.8% degradation efficiency in a mere 1.3-minute timeframe. This underscores the substantial impact of preventing EHP recombination in photocatalysts on achieving high photocatalytic efficiency.

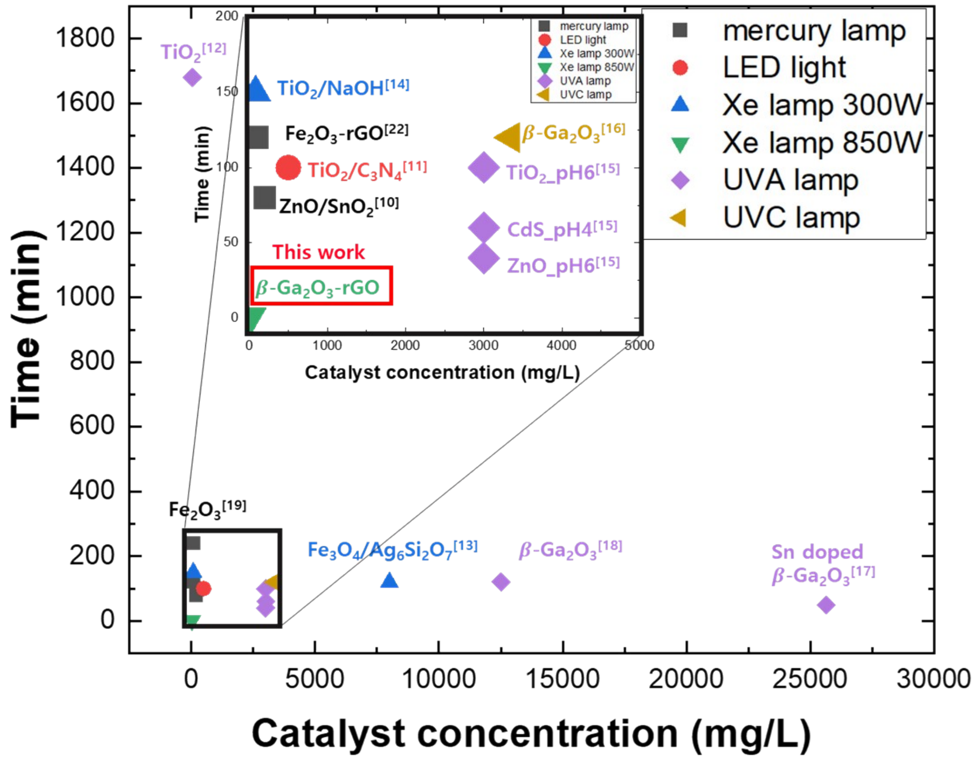

Fig. S6 Assessing various photocatalysts investigated for the degradation of methylene blue dyes.

Preparation of porous nanorod β-Ga_2O_3-rGO by hydrothermal synthesis

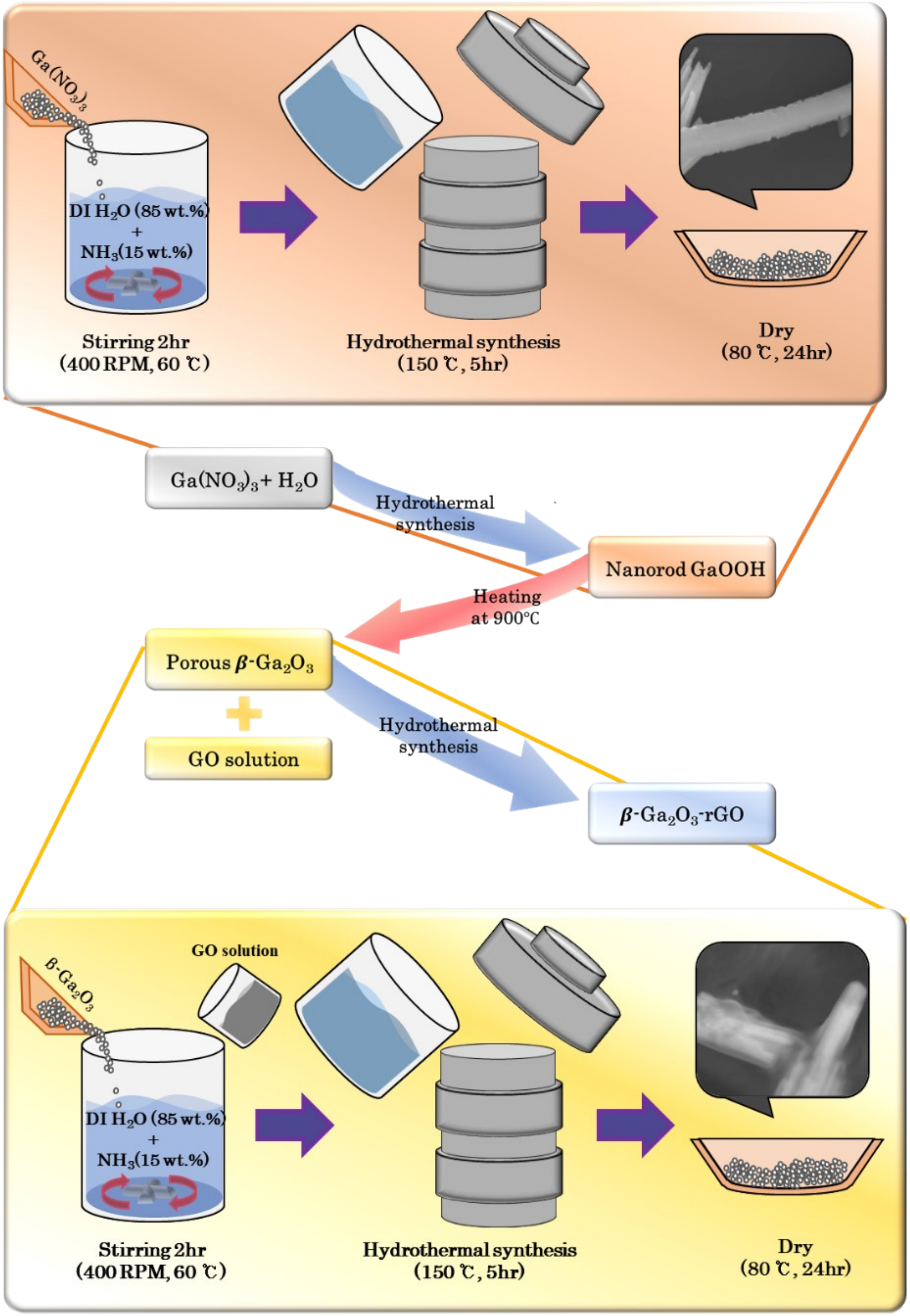

Fig. S7 Schematic image of β-Ga_2O_3-rGO synthesis process

References

(1) Park, S.; Ruoff, R. S. Chemical methods for the production of graphenes. *Nature nanotechnology* **2009**, *4* (4), 217-224.
(2) Luo, J.; Jang, H. D.; Sun, T.; Xiao, L.; He, Z.; Katsoulidis, A. P.; Kanatzidis, M. G.; Gibson, J. M.; Huang, J. Compression and aggregation-resistant particles of crumpled soft sheets. *ACS nano* **2011**, *5* (11), 8943-8949.
(3) Kim, J.; Cote, L. J.; Kim, F.; Huang, J. Visualizing graphene based sheets by fluorescence quenching microscopy. *Journal of the American Chemical Society* **2010**, *132* (1), 260-267.
(4) Tallinen, T.; Åström, J.; Timonen, J. The effect of plasticity in crumpling of thin sheets. *Nature materials* **2009**, *8* (1), 25-29.
(5) Lobkovsky, A.; Gentges, S.; Li, H.; Morse, D.; Witten, T. A. Scaling properties of stretching ridges in a crumpled elastic sheet. *Science* **1995**, *270* (5241), 1482-1485.
(6) Shenoy, S.; Chuaicham, C.; Shanmugam, M.; Okumura, T.; Balijapalli, U.; Li, W.; Balakumar, V.; Sasaki, K.; Sekar, K. Tailoring Interfacial Physicochemical Properties in Cu2O-TiO2@ rGO Heterojunction: Insights from EXAFS and Electron Trap Distribution Analysis. *ACS Applied Materials & Interfaces* **2023**, *15* (46), 54105-54118.
(7) How, G. T. S.; Pandikumar, A.; Ming, H. N.; Ngee, L. H. Highly exposed {001} facets of titanium dioxide modified with reduced graphene oxide for dopamine sensing. *Scientific reports* **2014**, *4* (1), 5044.
(8) Chen, D.; Zou, L.; Li, S.; Zheng, F. Nanospherical like reduced graphene oxide decorated TiO2 nanoparticles: an advanced catalyst for the hydrogen evolution reaction. *Scientific reports* **2016**, *6* (1), 20335.
(9) Woo, S.; Jung, H.; Yoon, Y. Real-Time UV/VIS Spectroscopy to Observe Photocatalytic Degradation. *Catalysts* **2023**, *13* (4), 683.
(10) Lin, J.; Luo, Z.; Liu, J.; Li, P. Photocatalytic degradation of methylene blue in aqueous solution by using ZnO-SnO2 nanocomposites. *Materials Science in Semiconductor Processing* **2018**, *87*, 24-31.
(11) Li, K.; Gao, S.; Wang, Q.; Xu, H.; Wang, Z.; Huang, B.; Dai, Y.; Lu, J. In-situ-reduced synthesis of Ti3+ self-doped TiO2/g-C3N4 heterojunctions with high photocatalytic performance under LED light irradiation. *ACS applied materials & interfaces* **2015**, *7* (17), 9023-9030.
(12) Kim, M. G.; Lee, J. E.; Kim, K. S.; Kang, J. M.; Lee, J. H.; Kim, K. H.; Cho, M.; Lee, S. G. Photocatalytic degradation of methylene blue under UV and visible light by brookite–rutile bi-crystalline phase of TiO 2. *New Journal of Chemistry* **2021**, *45* (7), 3485-3497.
(13) Chen, H.; Chen, N.; Gao, Y.; Feng, C. Photocatalytic degradation of methylene blue by magnetically recoverable Fe3O4/Ag6Si2O7 under simulated visible light. *Powder Technology* **2018**, *326*, 247-254.
(14) Hou, C.; Hu, B.; Zhu, J. Photocatalytic degradation of methylene blue over TiO2 pretreated with varying concentrations of NaOH. *Catalysts* **2018**, *8* (12), 575.
(15) Alkaim, A.; Aljeboree, A.; Alrazaq, N.; Baqir, S.; Hussein, F.; Lilo, A. Effect of pH on adsorption and photocatalytic degradation efficiency of different catalysts on removal of methylene blue. *Asian Journal of Chemistry* **2014**, *26* (24), 8445.
(16) Reddy, L. S.; Ko, Y. H.; Yu, J. S. Hydrothermal synthesis and photocatalytic property of β-Ga 2 O 3 nanorods. *Nanoscale Research Letters* **2015**, *10*, 1-7.
(17) Tien, L. C.; Chen, W. T.; Ho, C. H. Enhanced photocatalytic activity in β-Ga2O3 nanobelts. *Journal of the American Ceramic Society* **2011**, *94* (9), 3117-3122.
(18) Ananda, V. R.; Yulizar, Y. Green synthesis of β-Ga2O3 nanoparticles via Cnidoscolus aconitifolius leaf extract and photocatalytic activity for methylene blue degradation. *Solid State Sciences* **2023**, *139*, 107162.

(19) Bharath, G.; Anwer, S.; Mangalaraja, R.; Alhseinat, E.; Banat, F.; Ponpandian, N. Sunlight-Induced photochemical synthesis of Au nanodots on α-Fe2O3@ Reduced graphene oxide nanocomposite and their enhanced heterogeneous catalytic properties. *Scientific reports* **2018**, *8* (1), 5718.
